# A 20-year-old woman with pulmonary embolism and deep vein thrombosis associated with May-Thurner syndrome

**DOI:** 10.1016/j.radcr.2025.05.008

**Published:** 2025-06-05

**Authors:** Yusuke Yamazaki, Hidehiko Ikura, Toru Egashira, Toshimi Kageyama, Masaru Shibata, Kazunori Moritani, Hideo Mitamura

**Affiliations:** Department of Cardiology, Tachikawa Hospital, Tokyo, Japan

**Keywords:** May-Thurner syndrome, Iliac vein compression syndrome, Deep vein thrombosis, Pulmonary embolism, Woman, Computed tomography angiography, Duplex ultrasonography

## Abstract

May-Thurner syndrome (MTS) is a clinical condition characterized by the compression of the left common iliac vein between the overlying right common iliac artery and the underlying lumbar vertebrae. Although MTS can lead to deep vein thrombosis (DVT) and, in some cases, pulmonary embolism (PE), it remains underrecognized in clinical practice. We present a case of PE and DVT associated with MTS in a 20-year-old woman with a prolonged history of taking oral contraceptives (OCs). Initial computed tomography angiography (CTA) raised suspicion of MTS, which was further confirmed by Duplex ultrasonography. Anticoagulation therapy, along with discontinuation of OCs, resulted in resolution of thrombi in both the pulmonary and deep venous systems, with no recurrence following cessation of anticoagulation. This case underscores the importance of combining multiple imaging modalities in the evaluation of young women presenting with left-sided DVT, which eventually led to the diagnosis and proper management of MTS.

## Introduction

May-Thurner syndrome (MTS), also known as iliac vein compression syndrome, is typically defined as external compression of the left common iliac vein (LCIV) between the right common iliac artery (RCIA) and the lumbar spine [1,2]. It is more prevalent in women aged 20-40 years, with a female-to-male ratio ranging from 2:1 to 4.7:1 [3,4], and is often associated with the use of OCs, pregnancy, low body weight, or lumbar lordosis [[5], [6], [7], [8], [9]]. Although awareness of this condition has increased since it was first described by May and Thurner in 1957, it is still frequently overlooked due to its nonspecific clinical presentation [10]. DVT, as a complication of MTS, may occur in the presence of predisposing factors such as prolonged immobility, dehydration, or malignancy. However, these factors can also independently cause DVT, even in the absence of MTS. Therefore, identifying MTS as the primary underlying cause of DVT remains a diagnostic challenge without further detailed examination [10]. Given its potential for serious complications, early diagnosis and appropriate management of MTS are essential for clinicians treating DVT. Here, we report a case of pulmonary embolism (PE) and DVT associated with MTS in a young woman taking OCs. The diagnosis was established using computed tomography angiography (CTA) in conjunction with duplex ultrasonography, contributing to the subsequent management of the patient.

## Case presentation

**Fig. 1:**
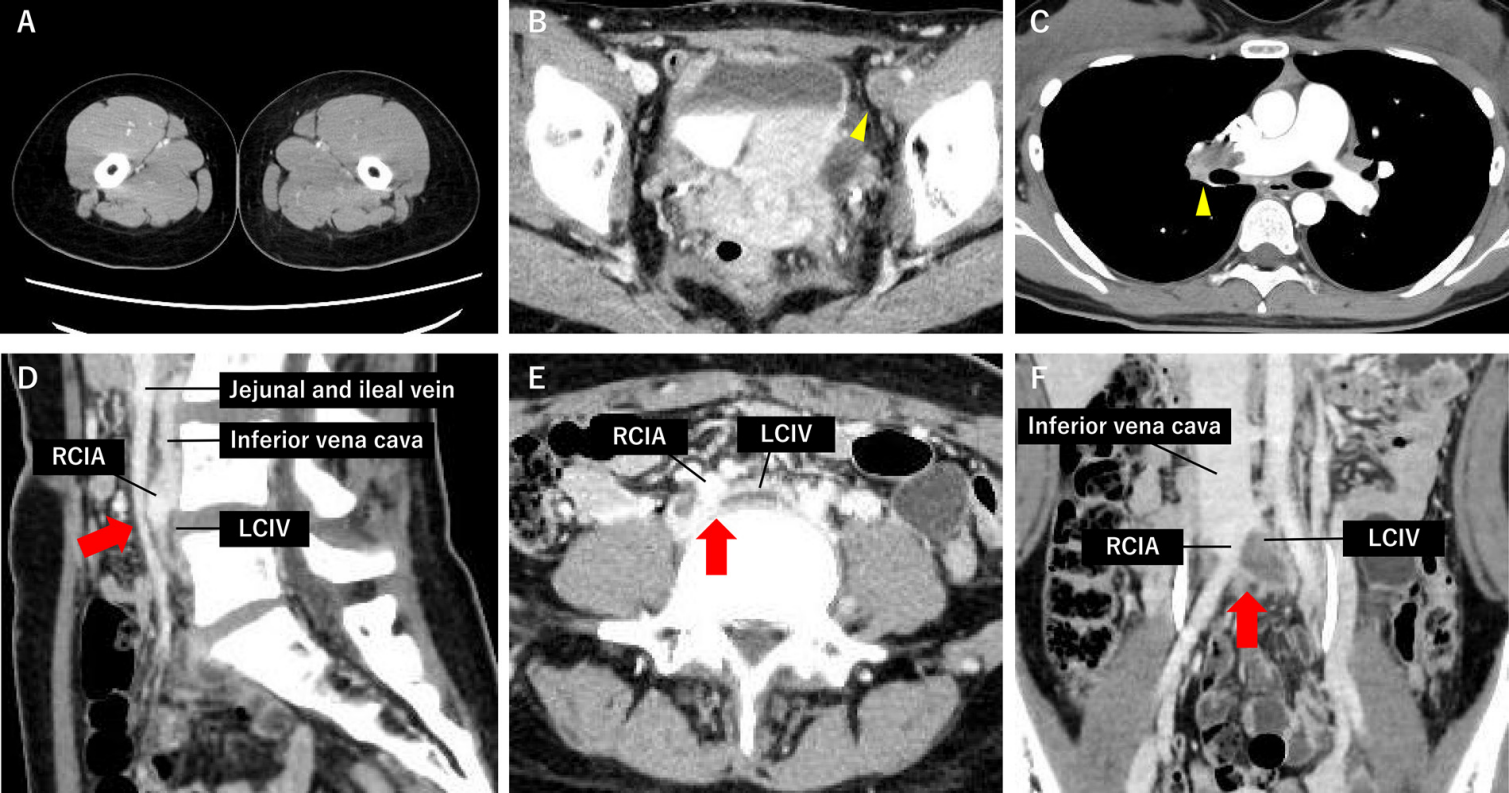
Initial CT angiography. (A) Left leg edema. (B) Left femoral venous thrombus (yellow arrowhead). (C) Right main pulmonary arterial thrombus (yellow arrowhead). (D and E) Left common iliac vein (LCIV) is compressed between the right common iliac artery (RCIA) and the fourth lumbar vertebra (red arrows). (F) Thrombi in the LCIV just distal to the compressive site by the crossing RCIA (red arrow).

A 20-year-old thin female presented to the emergency department with complaints of lightheadedness and chest tightness developing in a hot summer day. She also had a 1-month history of swelling in her left lower extremity (Fig. 1A). She had been taking OCs (drospirenone and ethinylestradiol) for approximately 3 years to manage her dysmenorrhea, and had no other significant medical history, such as alcohol or tobacco use, or childbirth. She reported insufficient fluid intake despite the hot summer weather. She denied any recent travel, trauma, surgery, or a family history of coagulopathies. Upon arrival, she was conscious, with a heart rate of 79 beats per minute, blood pressure of 93/66 mmHg, and peripheral oxygen saturation of 97% on room air. Her body mass index was 18.7 kg/m². Physical examination showed mild pitting edema in the left lower extremity. The pulmonary component of the second heart sound was accentuated, with no audible murmurs. Initial laboratory data showed a white blood cell count of 10,400/μL, hemoglobin level of 13.5 g/dL, platelet count of 216,000/μL, D-dimer level of 14.7 μg/mL, C-reactive protein of 2.43 mg/dL, Troponin I <0.010 ng/mL, and brain natriuretic peptide of 139.8 pg/mL. Electrocardiogram (ECG) demonstrated sinus rhythm with S1Q3T3, a typical McGinn-White pattern, and inverted T waves in the precordial leads [11]. Echocardiography showed right ventricular enlargement and a D-shaped left ventricle during systole. Tricuspid regurgitant jet velocity was 3.03 m/sec, indicating mild pulmonary hypertension. The left ventricular ejection fraction was preserved at 73%. Given her symptoms, unilateral leg edema, and these findings, PE secondary to DVT was suspected. Subsequent systemic CTA revealed thrombi in the bilateral pulmonary arteries and DVT in the left lower extremity (Fig. 1, Fig. 1). Notably, the thrombus in the LCIV was located just distal to the crossing of the RCIA, suggesting a possible involvement of MTS (Fig. 1, Fig. 1). Intravenous unfractionated heparin was initiated, and the patient was admitted to the high-care unit. Activated partial thromboplastin time (APTT) was tightly controlled with a therapeutic range of 70-90 seconds. Due to concerns regarding thrombosis, OCs were discontinued. Further evaluation yielded no evidence of the underlying cause of thrombophilia, including autoimmune diseases, inherited coagulopathies, or malignancy. Clinical and laboratory parameters gradually improved. On day 9, with a D-dimer level of 4.1 μg/mL, intravenous unfractionated heparin was switched to oral apixaban at a dose of 5 mg twice daily [12,13]. Follow-up CTA performed 2 weeks later showed improvement in both PE and DVT, although residual thrombi remained in the LCIV region. The patient was discharged on day 18 with improved ECG findings and D-dimer levels while apixaban administration was continued.

**Fig. 2:**
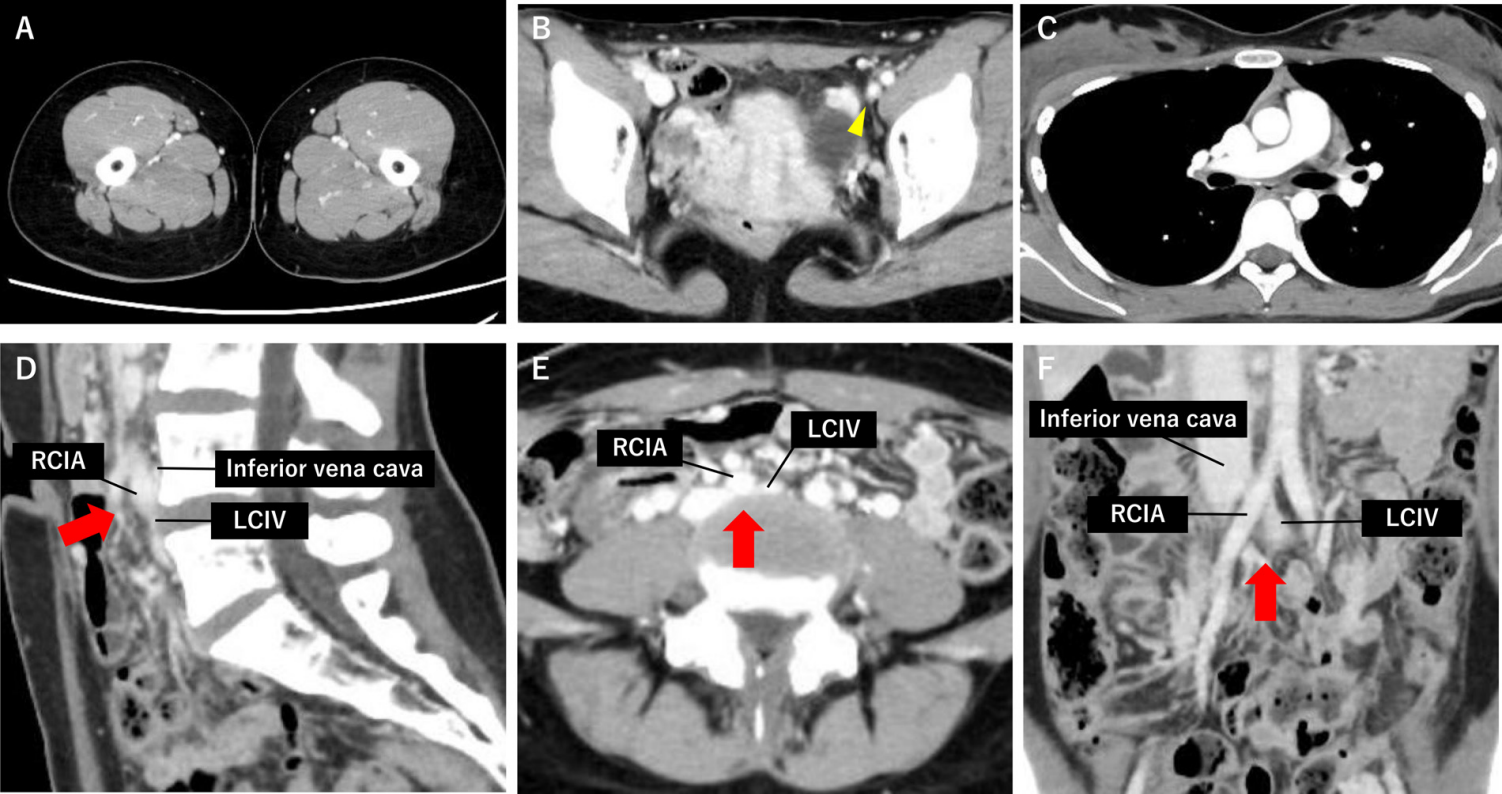
CT angiography performed after 2 months of anticoagulation therapy. (A) Improved left leg edema. C, Right pulmonary arterial thrombus is resolved. (B and D-F) There is a near-complete resolution of thrombi from the left common iliac vein (LCIV) to the common femoral vein (yellow arrowhead). Persistent LCIV compression between the right iliac common artery (RCIA) and the fourth lumbar vertebra is still evident (red arrows).

**Fig. 3:**
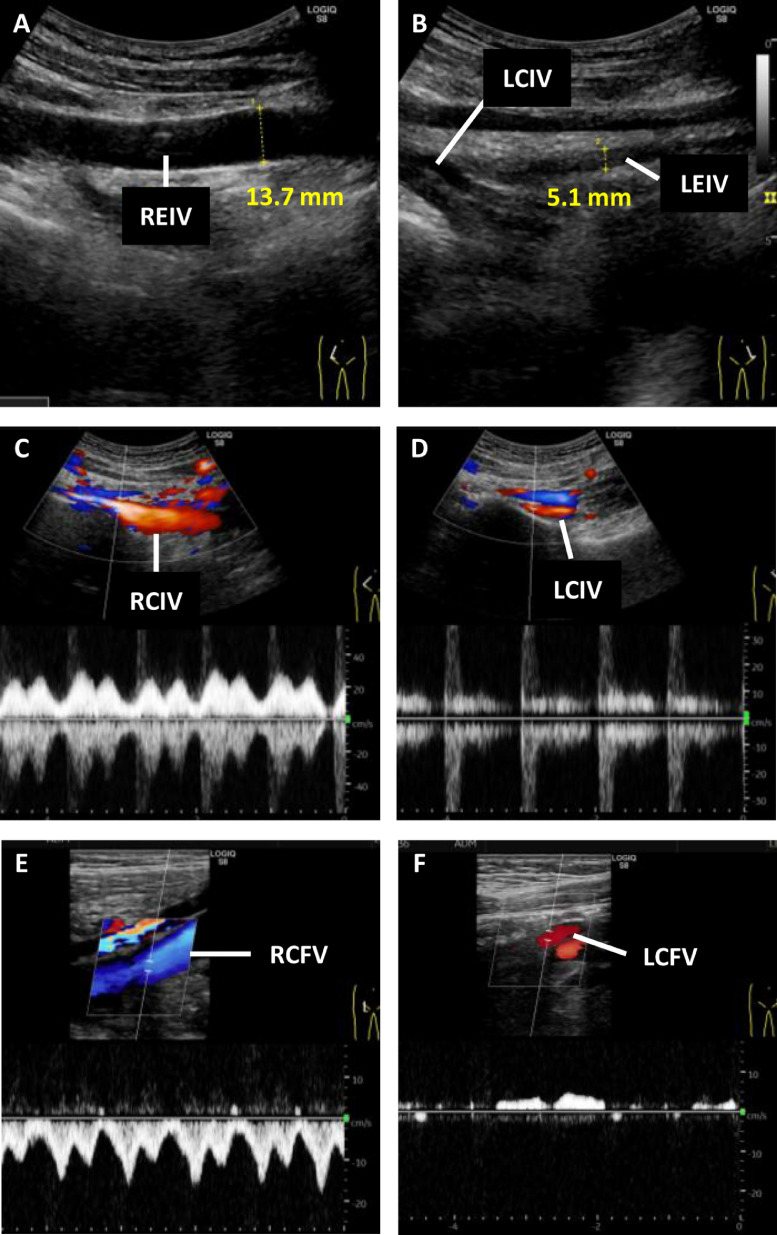
Duplex ultrasonography after resolution of deep vein thrombosis. (A and B) Transversal scan demonstrating that the diameter of the left external iliac vein (LEIV) is smaller than that of the right EIV (REIV). (C and D) Color Doppler ultrasound. The right common iliac vein (RCIV) shows normal phasic flow, while the left CIV (LCIV) shows a lack of phasic flow immediately distal to the site of compression by the right common iliac artery. (E and F) There is no venous reflux in the right common femoral vein (RCFV), whereas the left CFV (LCFV) shows reflux Doppler flow due to hemodynamic changes resulting from compression.

Two months postdischarge, follow-up CTA confirmed significant resolution of PE and DVT, even though the compression of LCIV by the RCIA against the fourth lumbar vertebra persisted (Fig. 2). Detailed examination with Duplex ultrasound revealed significant narrowing of the LCIV and left external iliac vein, with decreased Doppler flow (Fig. 3A and D). The LCIV lacked normal phasic flow compared to the right side (Fig. 3, Fig. 3), and reflux Doppler flow was observed in the left common femoral vein (Fig. 3, Fig. 3). Based on these imaging findings, the patient was diagnosed with MTS, most likely triggered by long-term OCs use and mild dehydration during summer. Since prolonged anticoagulation therapy and cessation of OCs led to marked thrombus resolution and symptom relief, more invasive interventions such as thrombolysis or endovascular procedures were deemed unnecessary. According to the patient’s preference and current national guidelines for DVT management, apixaban was discontinued 4 months after discharge as a part of a planned withdrawal of anticoagulation therapy [14]. Two months after discontinuing apixaban, the patient remained asymptomatic, with no leg edema or elevation in D-dimer levels. Follow-up CTA confirmed no recurrence of thrombus formation even without anticoagulant therapy. Therefore, we concluded that resuming anticoagulation therapy was unnecessary. She has remained off OCs and event-free under close clinical monitoring.

## Discussion

MTS is characterized by the compression of the LCIV between the RCIA and the lumbar vertebrae, as observed in the present case [1,2,5]. MTS is more prevalent among women [[3], [4], [5], [6]] and is typically associated with several precipitating factors, including the use of OCs, recent surgery, malignancy, dehydration, hypercoagulable states, and pregnancy [5,10,15,16]. Although MTS is often asymptomatic and tends to be underdiagnosed, timely recognition is crucial, as severe cases may require endovascular or surgical intervention.

Diagnosis of MTS is usually made using CT venography, MR venography, Duplex ultrasonography, catheter-based venography, or intravascular ultrasound, some of which are invasive in nature [5]. In this case, the initial CTA revealed thrombus formation at the crossing site of the LCIV and RCIA, making us suspect the involvement of MTS (Fig. 1). However, as DVT of the left lower extremity can also occur even in the absence of MTS, additional Duplex ultrasonography was performed after thrombus resolution, which confirmed the findings consistent with MTS. Due to its noninvasive nature, Duplex ultrasound is considered the first-line diagnostic measure for MTS. While this modality generally provides limited visualization of the common iliac vessels, diagnostically adequate imaging was obtained in this case [1,17].

Management of MTS typically involves 2 key components: treatment of the acute thrombotic event and correction of the underlying compression of the LCIV [1,18]. Anticoagulation therapy is the initial treatment for thrombotic MTS; however, in more severe cases, thrombolysis or mechanical thrombectomy may be required [19]. To prevent recurrence of thrombotic events, endovascular intervention, such as stent placement, may also be recommended [2,20]. In the present case, given the patient's young age and potential for future pregnancy, the option of endovascular intervention, which would necessitate long-term antithrombotic therapy, was carefully evaluated [19]. Importantly, our patient lacked chronic MTS-related history but had reversible factors such as poor hydration and the use of OCs, which may have contributed to temporary thrombus formation. Therefore, eliminating these exacerbating factors was expected to reduce the risk of recurrence. Indeed, following the cessation of OCs and implementation of adequate hydration guidance, along with approximately 4 months of anticoagulation therapy, the thrombus has resolved significantly. Moreover, we eventually decided to discontinue anticoagulation therapy in this young female patient with dysmenorrhea after weighing its merits and potential demerits of bleeding complications. In fact, even after 2 months off anticoagulation, there was no evidence of recurrence, with no elevation of the D-dimer level or thrombus formation in follow-up imaging studies, supporting the safety and appropriateness of discontinuing anticoagulation therapy in this patient. Whereas our approach reflected a thoughtful consideration of the patient’s potential future peripartum period, we believe that another special caution should be paid during any future pregnancy, when compression of the LCIV can become a matter of concern. The suspicion of underlying MTS involvement based on the initial CTA allowed us for early recognition of a higher thrombotic risk, enabling proactive discussion with the patient regarding future peripartum risks and other treatment options.

## Conclusions

This case highlights the importance of considering MTS in young women presenting with left lower extremity DVT, which can be suspected using a standard CTA but can be confirmed with additional Doppler ultrasonography after thrombus resolution. Particularly in young women, it is crucial to determine a treatment strategy based on the clinical background with careful consideration of potential future pregnancies.

## Data availability

Imaging and clinical data may be obtained by contacting the corresponding author.

## Patient consent

The patient provided written informed consent for the publication of the case report.
